# MicroRNA therapy confers anti-senescent effects on doxorubicin-related cardiotoxicity by intracellular and paracrine signaling

**DOI:** 10.18632/aging.203743

**Published:** 2021-12-05

**Authors:** Wenzheng Xia, Bowen Chang, Liqun Li, Tingting Hu, Jiaqi Ye, Hanbin Chen, Wenfeng Li, Tao Zan, Meng Hou

**Affiliations:** 1 Department of Plastic and Reconstructive Surgery, Shanghai Ninth People’s Hospital, Shanghai Jiao Tong University School of Medicine, Shanghai, Shanghai, China; 2 Department of Plastic Surgery, First Affiliated Hospital, Wenzhou Medical University, Wenzhou, Zhejiang, China; 3 Department of Neurosurgery, The First Affiliated Hospital of USTC, Division of Life Sciences and Medicine, University of Science and Technology of China, Hefei, Anhui, China; 4 Department of Radiation Oncology, First Affiliated Hospital, Wenzhou Medical University, Wenzhou, Zhejiang, China

**Keywords:** doxorubicin, cardiotoxicity, microRNA, senescence, exosome

## Abstract

Doxorubicin (Dox), an important anthracycline, is a potent anticancer agent that is used for treating solid tumors and hematologic malignancies. However, its clinical use is hampered by cardiac cardiotoxicity. This study aimed to investigate the cardioprotective potential of miR-199a-3p. Continuous Dox treatment not only markedly induced cardiomyocyte senescence but also resulted in a growing number of senescence-associated secretory phenotype (SASP) cardiomyocytes, frequently leading to heart senescence. This study showed that miR-199a-3p was downregulated in cardiomyocytes when exposed to Dox. The cardiac-specific overexpression of miR-199a-3p promoted cell cycle re-entry and cell proliferation, resulting in relief from cardiac senescence. Also, the elevation of miR-199a-3p inhibited the generation of SASP, thus, hampering the spread of senescence. In cardiomyocytes, the modulation of miR-199a-3p changed the levels of senescence-related protein GATA4. The ectopic expression of GATA4 blunted the anti-senescence effect of miR-199a-3p. Together, the data supported a role for miR-199a-3p during Dox cardiotoxicity. The elevation of miR-199a-3p might provide a dual therapeutic advantage in Dox cardiotoxicity therapy by simultaneously preventing cardiac senescence and reducing the spread of senescence.

## INTRODUCTION

Anthracyclines are among the most potent and widely prescribed chemotherapeutics since the last century and still are the cornerstones of cancer treatment in combination with new therapies [1]. However, the clinical use of these drugs, is restricted by cardiotoxicity, often suggesting modification or even discontinuation of potentially successful anticancer regimens [2]. Cardiotoxicity commonly happens within the first year after therapy completion, as lifespans are lengthened, thus elevating morbidity and mortality among cancer survivors [3].

Although clinical assessment allows the early detection of cardiotoxicity, validated prevention and treatment represent still-unmet clinical needs. Thus, an increasing number of oncologists have focused on optimal cardioprotective treatments and mechanisms [4]. A previous study suggested that Dox-related cardiotoxicity may result from cardiomyocyte senescence [5]. Demaria et al. has suggested that eliminating therapy-induced senescent cells alleviated several short- and long-term effects of the drugs, including cardiac dysfunction, bone marrow suppression, even cancer recurrence [6]. Persistent cellular senescence caused chromatin reorganization and acquisition of a secretome composed of cytokines, chemokines, and extracellular matrix factors, all known as the senescence-associated secretory phenotype (SASP) [7, 8]. Previous studies showed that senescent cardiomyocytes acquired an SASP that negatively affected healthy nonsenescent cardiomyocytes, rendering them senescent [9]. Ideally, optimal cardioprotective treatments should not only interfere with alleviating cardiomyocyte senescence but also reduce the spread of senescence.

MicroRNAs exhibited regulating entire gene expression networks and taking significant effect in cardiovascular diseases [10]. Although several studies have examined the regulation of cardiac senescence by microRNAs [11, 12], less is known about their functional roles in this context. Previously, functional screening identified many miRNAs that had the potential to exert an anti-senescent effect and induce cardiac regeneration [13]. Among these, miR-199a-3p, upon intra-cardiac injection, was shown to stimulate cardiomyocyte proliferation and promote cardiac regeneration [14, 15]. This study explored the therapeutic potential of miR-199a-3p in cardiac regeneration and protection against Dox-related cardiotoxicity.

GATA4 is one of the outstanding factors regulating both developmentally programmed senescence and damage-induced senescence [16]. GATA4, which is evolutionarily conserved, participates in an extensive range of biological processes, including cell cycle control, DNA damage, and energy metabolism, thus, contributing to tissue senescence [17, 18]. GATA4 serves as a prominent switch in the senescence regulatory network to activate SASP [16]. *In vivo* and *in vitro* models confirmed that GATA4 activated by SASP or other developmental cues regulated senescence via the upregulation of p16 in damage-induced and developmental senescence, which was an important factor in Dox-related cardiotoxicity [19]. These findings indicated that GATA4 took significant effect in senescence as one of the prominent sensors mediating senescent signaling in response to environmental stresses. Therefore, modulating this factor might alleviate Dox-related cardiotoxicity.

This study identified miR-199a-3p as a prominent determinant of anthracycline cardiotoxicity and provided a proof of concept that modulating miR-199a-3p/GATA4 was an incomparable means of preventing Dox cardiotoxicity, while inhibiting the spread of senescence. The genetic induction of miR-199a-3p elevation might provide a new approach against anthracycline-induced heart disease.

## RESULTS

### Dox-treated cardiomyocytes exhibited increased cellular senescence

**Figure 1:**
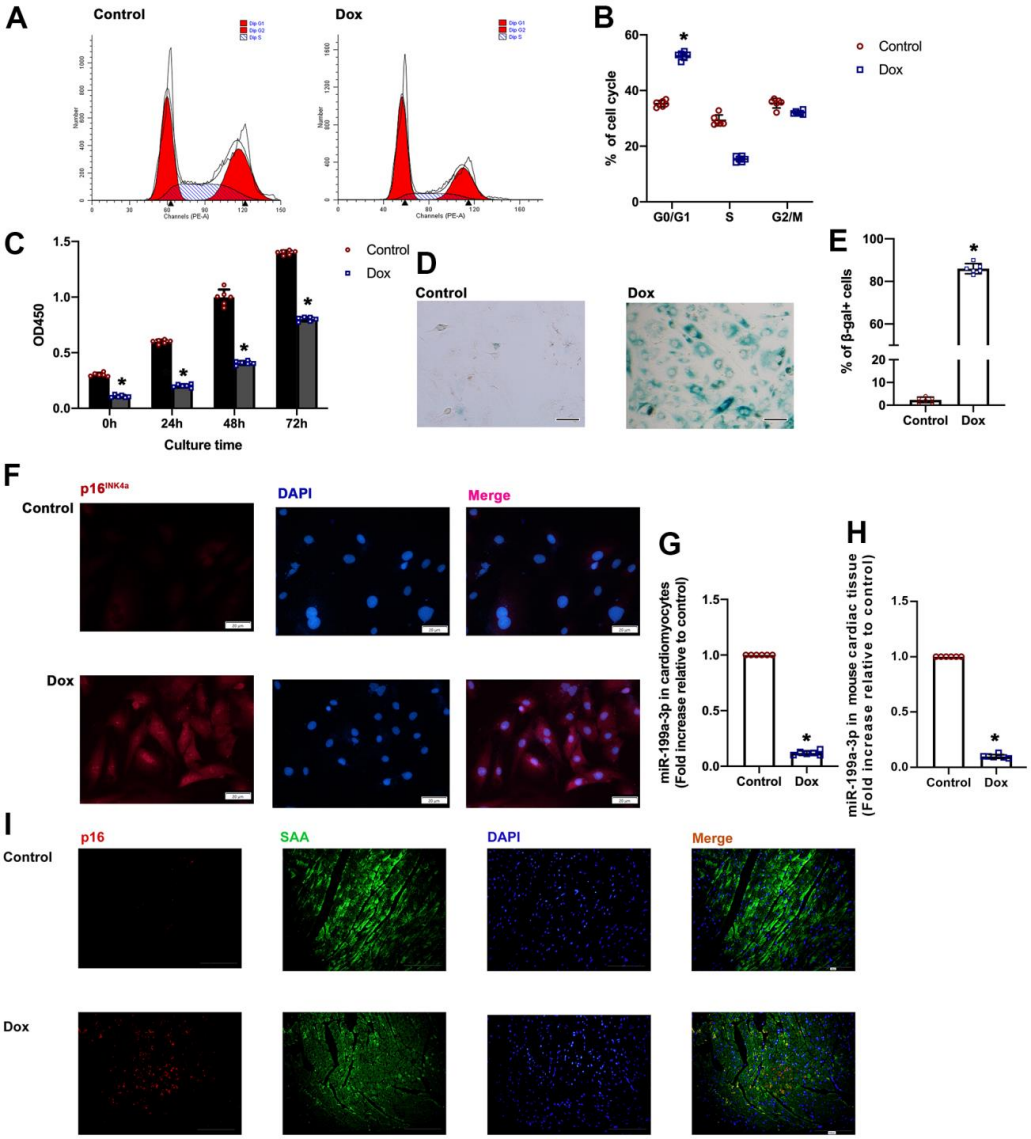
**Dox-treated cardiomyocytes exhibited increased cellular senescence.** (**A**) Cell cycle analysis was performed with PI staining by flow cytometry. (**B**) Percentages of cells in the three phases of the cell cycle. (**C**) Cellular proliferation was measured using CCK-8 assay. (**D**) Representative images of SA-β-gal staining (senescent cells are stained green). Scale bars, 20 μm. (**E**) Percentage of senescent cells was calculated. (**F**) Representative p16^INK4a^ staining (green). Scale bars, 20 μm. (**G**, **H**) Expression of miR-199a-3p in cardiomyocytes and cardiac tissue was quantified by qRT-PCR. ^*^P < 0.05 versus control, *n* = 6 per group. (**I**) Representative photomicrographs of p16^INK4a^-positive cardiomyocytes in the Dox treated cardiac tissue. Scale bars, 100 μm.

The flow cytometry analysis of the cellular cycle revealed an increased number of cardiomyocytes trapped in the G0/G1 phase in the Dox-treated group (Figure 1A, 1B). The cell growth curves showed that Dox-treated cardiomyocytes exhibited lower proliferative ability compared to control cells (Figure 1C). Furthermore, the cells in the Dox-treated group exhibited increased levels of senescence-associated β-galactosidase (SA-β-gal) activity (Figure 1D, 1E). Additionally, p16^INK4a^ immunostaining showed that cellular senescence was activated in the Dox-treated cardiomyocytes (Figure 1F). Taken together, these data showed that Dox-treated cardiomyocytes exhibited increased cellular senescence. Interestingly, the decreased level of miR-199a-3p was found in Dox-treated cardiomyocytes and mouse heart tissue, which was confirmed by qRT-PCR (Figure 1G, 1H). At meanwhile, more p16^INK4a^ -positive cells presented in the Dox -treated animals compared with the controls (Figure 1I).

### A slowdown of Dox-induced aging via the overexpression of miR-199a-3p

**Figure 2:**
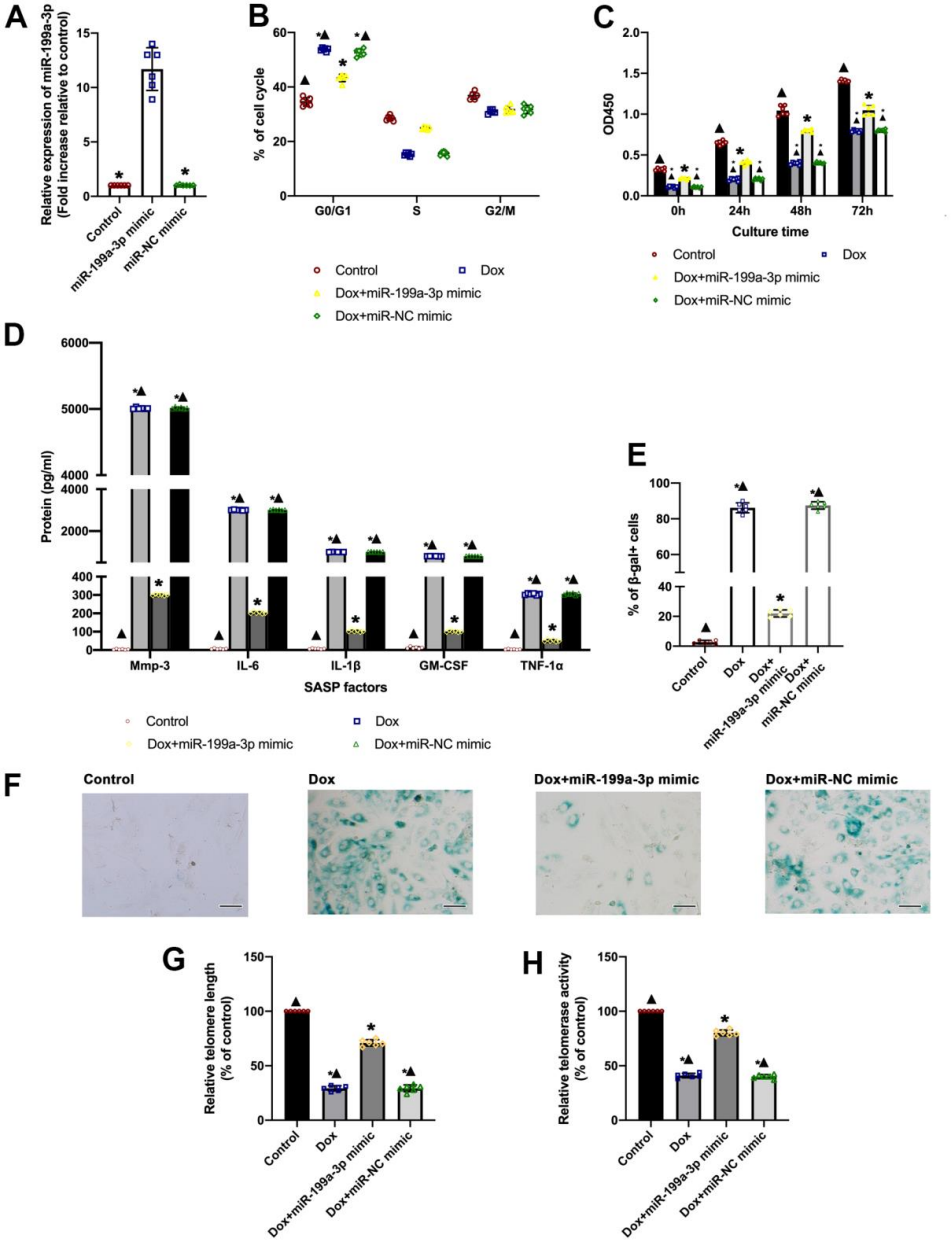
**A slowdown of Dox-induced aging via the overexpression of miR-199a-3p.** (**A**) Expression of miR-199a-3p was quantified by qRT-PCR. ^*^P < 0.05 versus the miR-199a-3p mimic in repeated-measures ANOVA, *n* = 6 per group. (**B**) Percentages of cells in the three phases of the cell cycle determined by flow cytometry. (**C**) Cellular proliferation was measured by the CCK-8 assay. (**D**) SASP factor protein levels quantified by Luminex of the medium. The medium was collected from such cells as follows: transfection with the miR-199a-3p mimic or miR-NC mimic, followed by treatment with Dox. The untreated cardiomyocytes were used as control. (**E**) The percentage of senescent cells was calculated. (**F**) Representative images of SA-β-gal staining (senescent cells are stained green). Scale bars, 20 μm. (**G**) Telomere length was detected by qRT-PCR. (**H**) Telomerase activity was determined using a telomerase repeat amplification protocol (TRAP). ^*^P < 0.05 versus control; ^▲^*P* < 0.05 versus Dox + miR-199a-3p mimic in repeated-measures ANOVA, *n* = 6 per group.

The study simultaneously investigated whether inducing miR-199a-3p could slow down the progression of aging. To do so, miR-199a-3p mimic transfection was performed to induce miR-199a-3p overexpression (Figure 2A). Hence, the study examined whether the elevation of miR-199a-3p in these Dox-treated cells might help overcome cellular senescence. Compared to the control cells, Dox-treated cardiomyocytes were distributed more in the G0/G1 phase, with impaired proliferative ability, while miR-199a-3p-overexpressing cardiomyocytes demonstrated a recovery in the cellular cycle and proliferation (Figure 2B, 2C). Then, SA-β-gal staining and SASP test were used to study aging-related physiology. Compared to the Dox-treated groups, the Dox+ miR-199a-3p mimic group displayed accelerated declines in SA-β-gal-positive cells and SASP (Figure 2D–2F). Telomere length and telomerase activity tests revealed that Dox-treatment caused a decline more than that in the control, while the elevation of miR-199a-3p slowed the speed of aging (Figure 2G, 2H). At meanwhile, latter after Dox treatment, cellular viability impaired and apoptosis happened, miR-199a-3p overexpression showed protective effect against apoptosis (Supplementary Figure 1A–1C).

### MiR-199a-3p directly targeted GATA4

**Figure 3:**
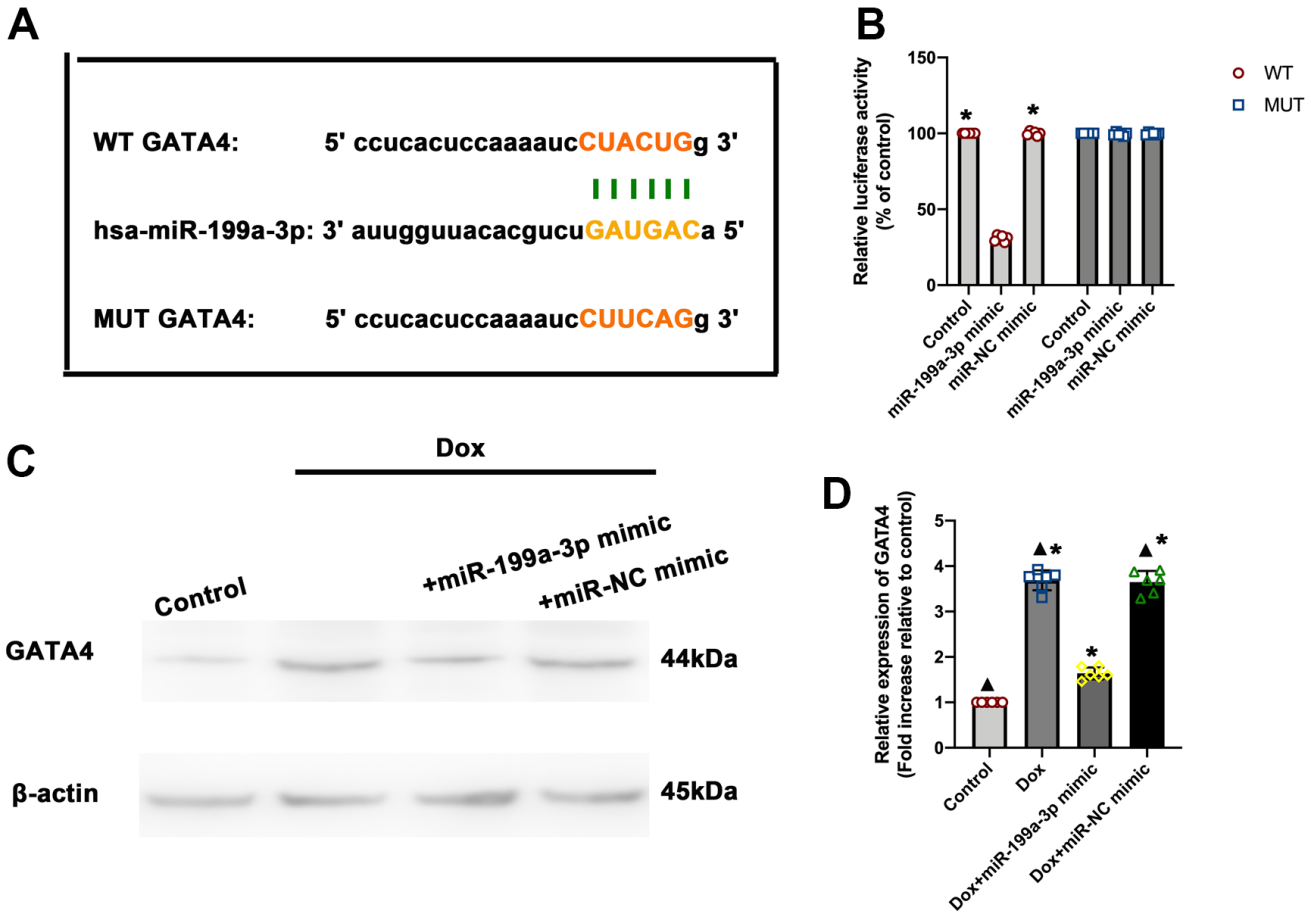
**MiR-199a-3p directly targeted GATA4.** (**A**) Predicted miR-199a-3p binding site in the 3’-UTR of GATA4. The corresponding sequence in the mutated (MUT) version is also shown. (**B**) Luciferase activity was analyzed 48 h after transfection of the miR-199a-3p mimic or miR-NC mimic. ^*^P < 0.05 versus the miR-199a-3p mimic (WT group) in repeated-measures ANOVA, *n* = 6 per group. (**C,** **D**) Western blot analysis of GATA4. The size of markers (in kDa) is indicated. ^*^P < 0.05 versus control; ^▲^*P* < 0.05 versus Dox + miR-199a-3p mimic in repeated-measures ANOVA, *n* = 6 per group.

TargetScan was used to predict the targets of miR-199a-3p. Among the predicted genes, GATA4 was chosen for further analysis, which was reported to participate in the heart senescence process [16]. To assess whether GATA4 was a direct target of miR-199a-3p, a 3’-UTR GATA4 reporter construct was used to assess the direct binding of miR-199a-3p to GATA4 mRNA. The overexpression of an miR-199a-3p mimic compared with control significantly decreased luciferase reporter activity, suggesting that miR-199a-3p bound directly to GATA4 mRNA (Figure 3A, 3B). The study also detected the protein of GATA4 in Dox, Dox + miR-199a-3p mimic, and Dox + miR-NC mimic groups. The GATA4 protein level was higher in the Dox group compared to the control, while it decreased in the Dox + miR-199a-3p mimic group (Figure 3C, 3D).

### MiR-199a-3p repressed GATA4 contributing to the anti-senescent effect against Dox

**Figure 4:**
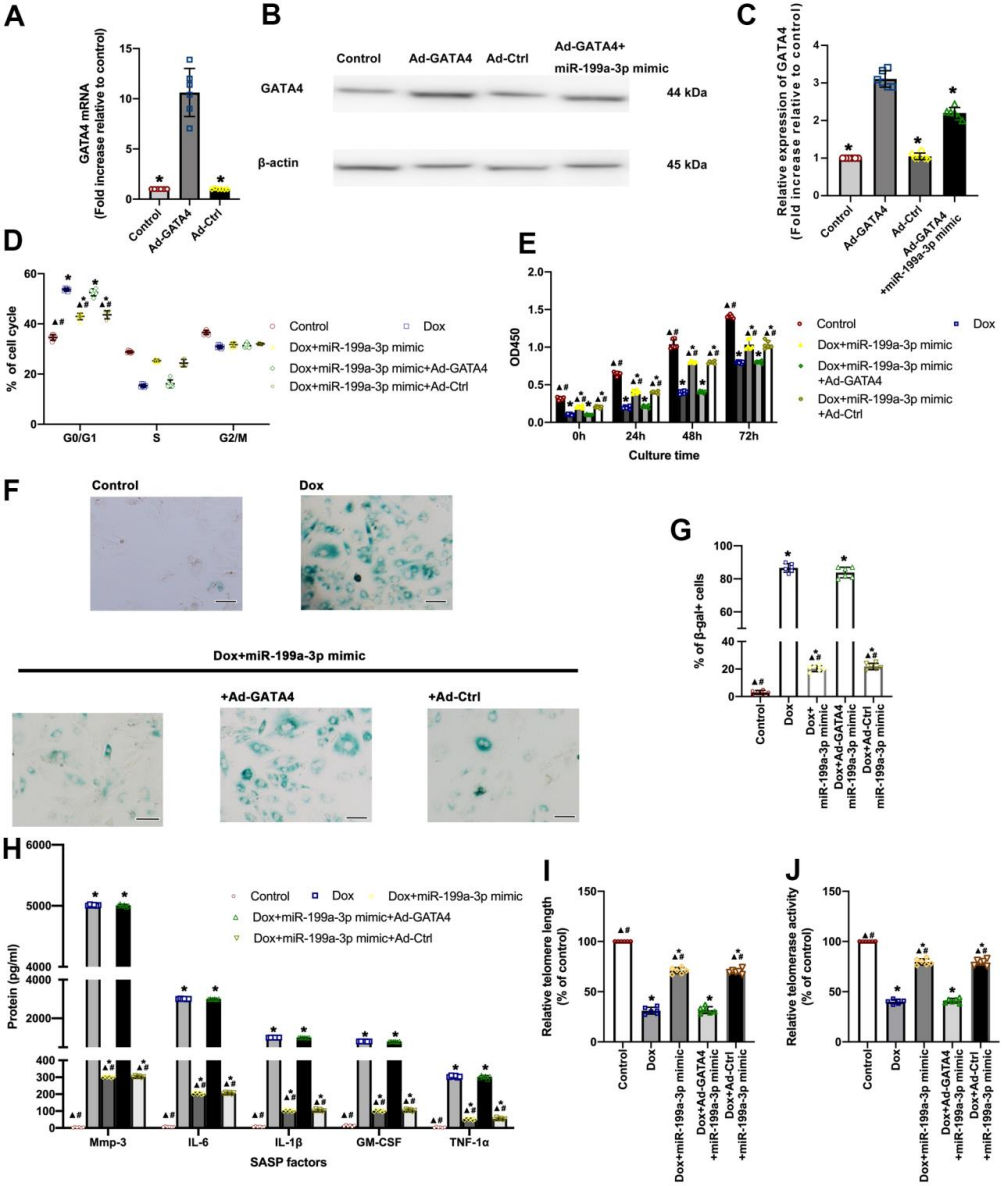
**MiR-199a-3p repressed GATA4 contributing to the anti-senescent effect against Dox.** (**A**–**C**) Cardiomyocytes were infected with Ad-GATA4, Ad-Ctrl, or Ad-GATA4+miR-199a-3p mimic. After 24 h, the cell lysates were harvested for qRT-PCR (**A**) and western blot analysis (**B,** **C**). ^*^P < 0.05 versus Ad-GATA4 in repeated-measures ANOVA, *n* = 6 per group. (**D**) The percentages of cells in the three phases of the cell cycle determined by flow cytometry. (**E**) Cellular proliferation was measured using the CCK-8 assay. (**F**) Representative images of SA-β-gal staining (senescent cells are stained green). Scale bars, 20 μm. (**G**) The percentage of senescent cells was calculated. (**H**) SASP factor protein levels quantified by Luminex of the medium. The medium was collected from such cells as follows: transfection with the miR-199a-3p mimic or the miR-199a-3p mimic + Ad-GATA4, or the miR-199a-3p mimic + Ad-Ctrl, followed by exposure to Dox. The untreated cardiomyocytes were used as the control. (**I**) Telomere length was detected by qRT-PCR. (**J**) Telomerase activity was determined using telomerase repeat amplification protocol (TRAP). ^*^P < 0.05 versus control; ^▲^*P* < 0.05 versus Dox; ^#^*P* < 0.05 versus Dox + miR-199a-3p mimic + Ad-GATA4 in repeated-measures ANOVA, *n* = 6 per group.

Ad-GATA4 was designed to induce the overexpression of GATA4 so as to test whether the anti-senescent effect of this miRNA in Dox-treated cardiomyocytes could be due to the inhibition of GATA4 (Figure 4A–4C). Through co-infection with the miR-199a-3p mimic and Ad-GATA4, it was confirmed that this approach led to the reduced anti-senescent effect of miR-199a-3p against Dox. For comparison, cell cycle and cellular proliferation were analyzed. Dox trapped more cells in the G0/G1 phase and inhibited proliferation, while miR-199a-3p relieved this. This anti-senescent effect was reversed by the co-infection with miR-199a-3p mimic and Ad-GATA4 (Figure 4D, 4E). The maintenance of GATA4 levels in Dox-treated cardiomyocytes preserved SA-β-gal+ and SASP cardiomyocytes, which was decreased by miR-199a-3p mimic (Figure 4F–4H). Of note, GATA4 overexpression was effective in reducing telomere length and telomerase activity, which was recovered by miR-199a-3p mimic transfection against Dox (Figure 4H, 4I).

### MiR-199a-3p eliminated the spread of senescence among cardiomyocytes

**Figure 5:**
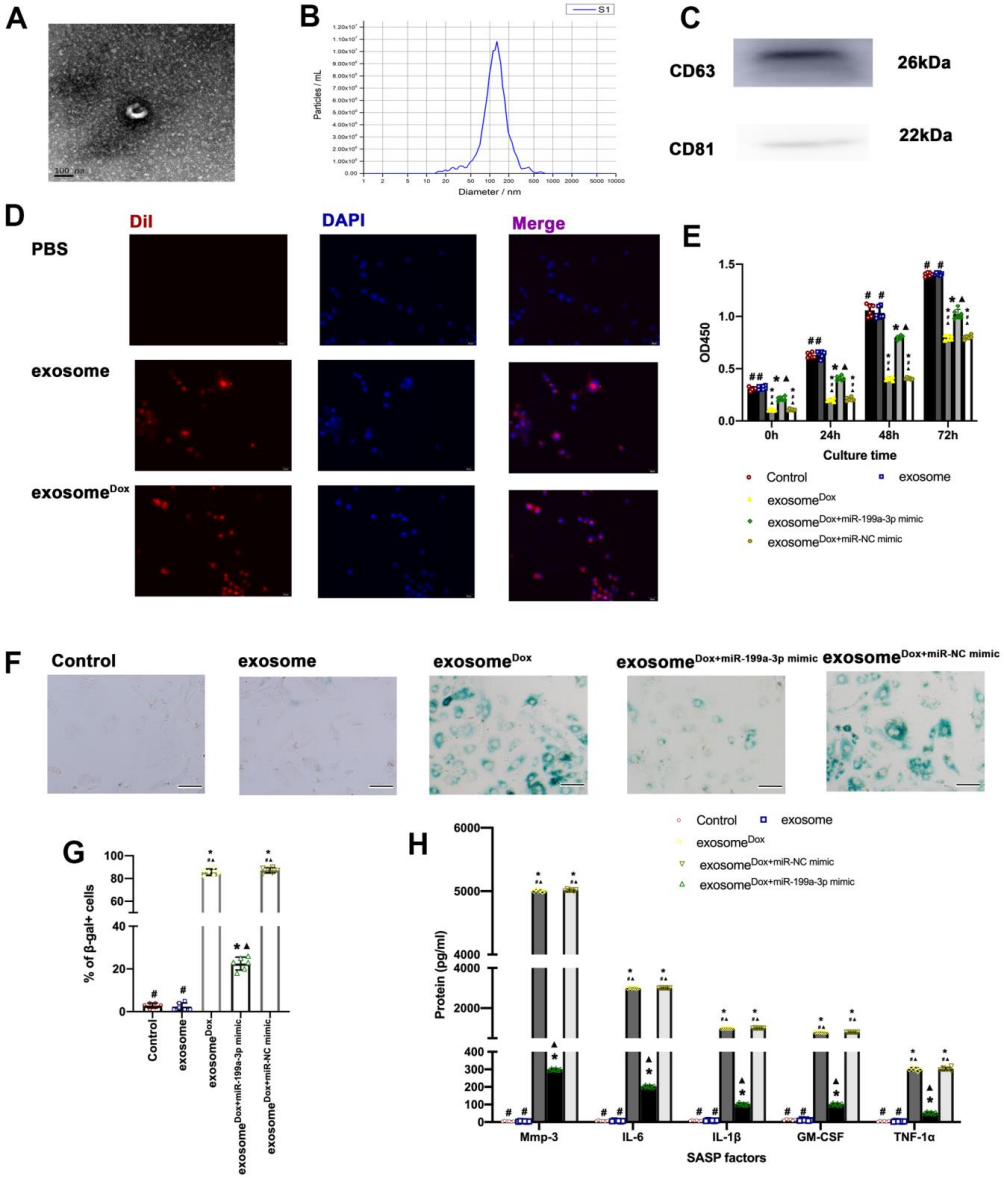
**MiR-199a-3p eliminated the spread of senescence among cardiomyocytes.** (**A**) Representative electron micrograph of isolated exosomes. Scale bar: 100 nm. (**B**) Size of exosomes measured using a Zetasizer Nano ZS instrument. (**C**) Protein levels of CD63 and CD81, two exosome markers. (**D**) DiI-labeled exosomes (red) were internalized into DAPI-labeled cardiomyocytes (blue). Scale bar, 20 μm. (**E**) Cellular proliferation was measured using the CCK-8 assay. (**F**) Representative images of SA-β-gal staining (senescent cells are stained green). Scale bars, 20 μm. (**G**) The percentage of senescent cells was calculated. (**H**) SASP factor protein levels quantified by Luminex of the medium. The medium was collected from cells as follows: treated with exosome, exosome^Dox^, exosome^Dox+miR-199a-3p mimic^, and exosome^Dox+miR-NC mimic^. The untreated cardiomyocytes were used as control. ^*^P < 0.05 versus control; ^▲^*P* < 0.05 versus exosome; ^#^*P* < 0.05 versus exosome^Dox+ miR-199a-3p mimic^ in repeated-measures ANOVA, *n* = 6 per group.

A subset of Dox-treated cardiomyocytes expressed SASP factors, and the SASP factors were inhibited by miR-199a-3p overexpression. Therefore, the present study explored whether miR-199a-3p overexpression could inhibit the spread of senescence among cardiomyocytes. To this end, exosomes were collected from cardiomyocytes (Figure 5A–5C), DiI staining showed that exosomes were swallowed by cardiomyocytes (Figure 5D). Exosomes derived from Dox-treated cardiomyocytes (exosome^Dox^) caused the development of senescence, accompanied by impaired proliferation (Figure 5E) and more SA-β-gal+ cells (Figure 5F, 5G). The extent of SASP secretion from exosome^Dox^-treated cardiomyocytes varied (Figure 5H). Notably, exosomes derived from miR-199a-3p + Dox-treated cardiomyocytes (exosome^Dox+miR-199a-3p mimic^) failed to induce the spread of senescence compared with exosome^Dox^ (Figure 5D–5H).

## DISCUSSION

The present study revealed miR-199a-3p as an important player of anthracycline cardiotoxicity and proposed miR-199a-3p overexpression as an effective methods of preventing the cardiac adverse effects of Dox, besides inhibiting the spread of senescence.

Although major efforts in understanding the mechanism of anthracycline-induced cardiotoxicity, the molecular and cellular details are still not yet fully uncovered [20, 21]. Potential mechanisms include the generation of ROS, causing DNA damage and mitochondrial dysfunction, thus, leading to cardiac senescence [1, 22]. One common character of senescent cells is an essentially irreversible cell cycle arrest, with quiescence of cell proliferation [23]. The present study revealed that more cells were trapped in the G0/G1 phase, with impaired cellular proliferation, when subjected to Dox. Senescent cells secrete many factors, including pro-inflammatory cytokines, chemokines and MMPs, collectively termed the SASP [24]. SASP constitutes a hallmark of senescent cells and mediates pathophysiological effects [25]. For example, the SASP reinforces and spreads senescence; meanwhile, SASP factors mediate developmental senescence [26, 27]. The results supported the notion that Dox not only caused cellular senescence but mediated the spread of senescence. A previous study raised the hope that inhibiting the spread of senescence could provide significant benefits in cardiac tissue regeneration [9], suggesting that it might be used to treat Dox-related cardiotoxicity. The findings showed that the alleviation of Dox-induced cardiomyocyte senescence could inhibit SASP, thus triggering a negative feedback loop and eventually inhibiting the spread of senescence.

A full understanding of the mechanism of Dox-related cardiac senescence remains elusive; however, the present study highlighted the role of miR-199a-3p in this process. Although miR-199a-3p expression has been associated with various disorders [28, 29], its precise functional role in cardiac diseases is still controversial. Minae An et al. reported that miR-199a-3p promoted cardiomyocyte growth and electrical activity, which in turn promoted electrical recovery and cardiac regeneration [30]. Anna Baumgarten et al. revealed that miR-199 was downregulated in dilated cardiomyopathy, thus, contributing to the loss of cardiac mass during the dilatation of the heart in the senescence process [31]. Keiichi Koshizuka et al. suggested that the miR-199 family inhibited cancer cell migration and invasion [32]. Such function can be used to identify a potential treatment for Dox-related cardiotoxicity, which meets the standard of the optimizing cardiac protective treatments in terms of not only interfering with the primary mechanisms of cardiotoxicity but at least preserving or even enhancing the antitumor efficacy of chemotherapy. Using the gain-of-function genetic approach in Dox-injured cardiomyocytes, it was found that the miR-199a-3p promoted cardiomyocyte proliferation and de-repressed cell cycle, thus, facilitating the recovery from senescence. These results concluded that the upregulation of miR-199a-3p might serve as an important regulatory component of a molecular pathway that alleviated the Dox-induced senescence. Meanwhile, exosomes and their miR cargo have been reported to act during intercellular communication in the cellular senescence process [33, 34]; miRs are packaged into exosomes to modulate cellular senescence and organismal aging [35]. Interestingly, the results revealed that exosome derived from Dox- treated cardiomyocytes induced senescence communication, while the spread of senescence was alleviated by miR-199a-3p overexpression.

MiR-199a-3p, same as other microRNAs, engages a broad series of mRNA targets, including numerous survival factors and cell cycle regulatory proteins to take effects [36, 37]. Thus, the potential promotion of the regenerative response by the miR-199a-3p involves the actions of protein targets that participate in cellular senescence [14]. The current study design pointed to an anti-senescence contribution of the miR-199a-3p via modulating GATA4. GATA4, as a key regulator of SASP and senescence, accumulates during cellular senescence [38, 39]. The SASP is manipulated by enhancer remodeling and activation of transcription factor GATA4 [16]. During the senescence process, this accumulated GATA4 initiates a transcriptional circuit to activate SASP [18]. In agreement, the findings revealed that Dox induced GATA4 accumulation, with the activation of SASP cardiomyocytes. In the aged chondrocytes, the inhibition of GATA4 suppressed SASP factors, leading to the relief from senescence [40]. In similarities with this view, the findings of the present study showed that the prompt inhibition of GATA4 by miR-199a-3p ensured a general anti-senescence effect against Dox-related toxic effects.

Some limitations existed in the current study. The hiPSC-derived cardiomyocytes were applied to investigate the role of senescence in Dox cardiotoxicity, this may not comprehensive enough. Further investigation is required to explore the key experiments replicated in primary cardiomyocytes, which are post-mitotic and non-replicating cells.

## CONCLUSIONS

Overall, miR-199a-3p might improve the prognosis of patients with cancer treated with Dox by concomitantly alleviating cardiomyocyte senescence as well as preventing the spread of senescence. Therefore, miR-199a-3p might eventually help “kill two birds with one stone” in patients with cancer requiring anthracycline chemotherapy.

## MATERIALS AND METHODS

### Animals

Male C57/Bl6 mice were maintained in accordance with the guidelines published by the US National Institutes of Health. During the experiments, mice were anesthetized with 1.5–2% isoflurane and kept warm on a heated platform. After the experiments, mice were sacrificed by CO_2_ inhalation.

### Cell culture and treatment

### Human-induced pluripotent stem cell–derived cardiomyocytes

Human-induced pluripotent stem cell–derived cardiomyocytes were purchased from Cellular Dynamics International (WI, USA). The cells were cultured as previously described [5].

### Dox treatment

The cardiomyocytes were cultured with Dox to model cardiac injury. The concentration of Dox was set at 0.5 μM and the exposure time at 24 h, as described previously [5].

### Cell cycle assay

Further, 70% cold anhydrous ethanol was used to fix the cells. Then, the cells were treated with propidium iodide (PI) (Sigma, MO, USA) and RNase A. A flow cytometer equipped with Cell Quest software was used to detect cell cycle distribution, as described previously [41].

### Cell proliferation assay

The cell counting kit-8 (CCK-8) assay was applied to estimate the cellular proliferation rate following the manufacturer’s protocol. Briefly, the CCK-8 solutions were added in the cells grown in a 96-well plate, following incubated with for 1 h at 37° C, following which the absorbance of each well at 450 nm was recorded.

### Senescence-associated β-galactosidase staining (SA-β-gal staining)

SA-β-gal staining was performed using a kit (Cell Signaling Technology, MA, USA) following the manufacturer’s protocols. Senescent cardiomyocytes were identified as green-stained cells under light microscopy. Total cells were counted in three random fields per culture dish to determine the percentage of SA-β-gal-positive cells [42].

### Immunofluorescence staining

Fixed with 4% PFA, the cardiomyocytes were treated with Triton X-100 at room temperature, blocked with 3% goat serum at 37° C, lasting for 1 h, and incubated with a primary antibody against p16^INK4a^ (ab108349, 1:50) at 4° C overnight. The cells were subsequently incubated with secondary antibodies conjugated with Alexa Fluor 594, followed by DAPI staining. The images were captured using a Leica fluorescence microscope. The calculations were conducted in a blinded manner.

Immunofluorescent staining was performed with paraffin-embedded tissues. Primary antibodies used in this study included those against Cdkn2a (p16^Ink4a^) (Abcam, ab211542), and Sarcomeric-α-actin (SAA; Invitrogen, 53-9760-82). Following primary incubation, slides were washed with 1X PBS and incubated with Alexa Fluor 594 (1:50; Invitrogen, A32740) secondary for one hour at room temperature. Sections were counterstained with DAPI staining medium and analyzed. Fluorescence was detected under a microscope.

### Quantitative reverse transcription–polymerase chain reaction

Total RNA was extracted with TRIzol reagent (Invitrogen, CA, USA) and then subjected to a reverse transcription reaction using a First-Strand cDNA Synthesis kit (Roche). cDNA was used for real-time quantitative polymerase chain reaction (Q-PCR) analysis. Data were normalized by the level of U6, or GAPDH expression, in each sample.

### Transfection with miR-199a-3p mimic

The miRNA mimic of miR-199a-3p was purchased from Invitrogen. The mirVana miRNA mimic was used to induce miR-199a-3p overexpression. Cardiomyocytes were transfected with a final concentration of 10 nmol/L for the miR-199a-3p mimic using an X-treme transfection reagent (Roche Applied Science, Penzberg, Germany) following the manufacturer's protocol.

### Luminex assay

The levels of cytokines, chemokines, growth factors, and matrix metalloproteinases (MMPs) were measured in multiplex assays with the Luminex bead-based platform and software. Luminex assays were performed on 50 μL of cellular medium, according to the product instructions, as previously described [43]. After the cellular medium was collected, viable cell counts were determined from each sample to normalize data.

### Relative telomere length measurement

Relative telomere length measurement in cardiomyocytes was performed using the PCR approach based on a previously established method [44], using Gapdh as the normalizing gene.

### Relative telomerase activity measurement

The telomerase activity of cardiomyocytes was examined using a Telo TAGGG Telomerase PCR ELISA Plus kit following the manufacturer’s protocols, as described previously [45].

### Luciferase assay

Luciferase assays were conducted via cotransfection with a 3’ -UTR-luciferase reporter plasmid and miRNA mimics (10nM) using Lipofectamine 2000 (Invitrogen). Luciferase activity was tested 48 h after transfection with a dual-luciferase reporter assay system (Promega, WI, USA) following the manufacturer’s protocol.

### Western blot analysis

Western blot analysis was conducted as previously described [46]. Primary antibodies, including GATA4 (ab256782, 1:1000), CD63 (ab59479, 1:750), and β-actin (ab179467) were purchased from Abcam. CD81 (#56039, 1:500) was purchased from Cell Signaling Technology.

### Transient transfection

To overexpress of GATA4, the cardiomyocytes were transduced with adenoviral GATA4 (Ad-GATA4) or adenoviral control (Ad-Ctrl), as described previously [47]. The qRT-PCR and western blot analysis were conducted to confirm the transfection efficiency.

### Isolation and tracing of exosomes

The exosomes were isolated and purified from the supernatants of cardiomyocytes. After 48 h of culture, the supernatants were collected. The exosome quick extraction solution was added to the filtered solution at a 1:5 ratio and stored at 4° C for at least 12 h. The characterization of exosomes was carried out as previously reported [46].

With respect to exosome tracing, the exosomes were labeled with DiI, followed by exosome isolation. With respect with the *in vitro* tracing of exosomes, DiI-labeled exosomes were incubated with the un-treated cardiomyocytes for 3 h. The cell nuclei were stained with DAPI, at a concentration of 1:1000 for 10 min at 37° C, as previously described [48]. The fluorescence was detected under a microscope.

### Statistical analysis

Data were expressed as the mean ± standard deviation. Statistical significance of differences between groups was tested by repeated-measures analysis of variance (ANOVA). The Student’s *t* test was used to compare the two groups. A *P*-value <0.05 indicated a statistically significant difference.

## Supplementary Material

Supplementary Figure 1
