# Supplementary material for: MicroRNA therapy confers anti-senescent effects on doxorubicin-related cardiotoxicity by intracellular and paracrine signaling
Source: Aging (Albany NY). 2021 Dec 5;13(23):25256–70. doi: 10.18632/aging.203743 (PMC8714172; doi:10.18632/aging.203743)
Supplement: Supplementary Figure 1 [file aging-13-203743-s001.pdf]

SUPPLEMENTARY FIGURE

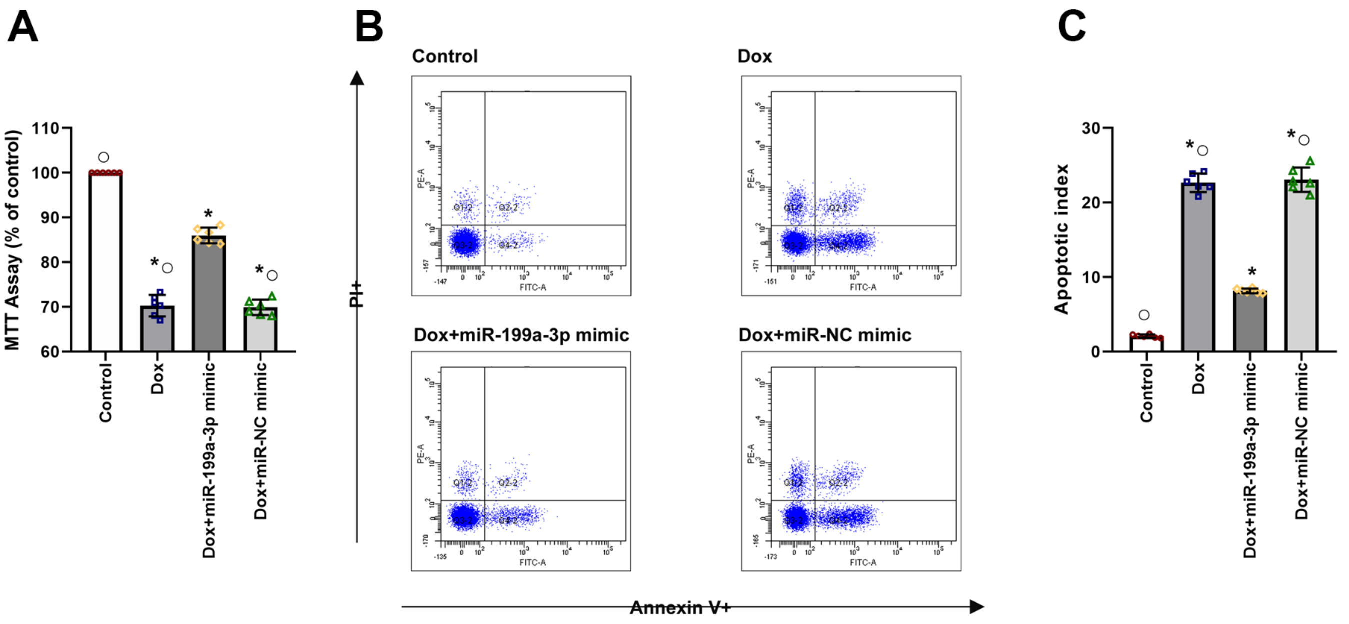

Supplementary Figure 1. Overexpression of miR-199a-3p took cellular protective effects against Dox. (**A**) Cell viability was analyzed with the MTT assay. (**B**, **C**) Representative flow cytometric dot plots of apoptotic cells after Annexin V/propidium iodide staining. $^{*}P < 0.05$ versus Control; $P < 0.05$ versus Dox +miR-199a-3p mimic in repeated-measures ANOVA, $n = 6$ per group.
